# Terahertz Plasmonic Field-Induced Conductivity Modulation in Gold

**DOI:** 10.1038/srep10812

**Published:** 2015-06-10

**Authors:** A. Y. Elezzabi, P. Maraghechi, S. R. Greig

**Affiliations:** 1 Ultrafast Optics and Nanophotonics Laboratory, Department of Electrical and Computer Engineering, University of Alberta, Edmonton, AB, T6G 2V4 Canada

## Abstract

We report the observation of terahertz (THz) electric field induced conductivity modulation in sub-wavelength gold plasmonic media. Through all-THz pump-probe time-resolved transmission spectroscopy, we demonstrate that the presence of induced surface charges influences near-field mediated light propagation. The phenomenon is ascribed to the enhanced metal conductivity due to enhanced surface density of conduction electrons. The surface induced charge dynamics are revealed via phase-dependent time-resolved signatures. The phenomenon is a prelude to a wide class of ultrafast active THz plasmonic devices and paves the way for plasmonic field effects devices, similar to semiconductor ones.

The behaviour of free and bound electrons in solids is one of the most fundamental areas of study in condensed matter physics. Understanding many critical, intricate mechanisms of interaction of electrons within materials is often revealed by introducing an external electric field. Electric field effects on material electronic properties are observed in nonmetallic media. Electrical conductivity modulation, Δσ, by an electric field, serves as core for many semiconductor electronic devices^1^. Particularly, electric field conductivity modulation of a field effect transistor channel is the heart of modern day high-speed semiconductor electronics. In these classes of devices, the electric field varies the charge concentration in the semiconducting channel and, subsequently, controls the amount of electrical current passing through it. On the contrary, for metals, the electric field induced Δσ is extremely imperceptible. This is due to the presence of a high density (*N*_*0*_ ~ 10^23^ cm^−3^) of conduction electron screening. A small DC conductivity modulation of Δσ ≈ 0.1% from a 10 nm thick Au film at an electric field of 10^6^ V/cm, was first reported by Bonfiglioli *et al*.^2^,^3^,^4^. Following experiments by Stadler^5^, showed a slightly higher Δσ ≈ 2% at 3 × 10^8^ V/cm. In these pioneering experiments, the electrical conductivity, σ, was shown to vary linearly with the electric field-induced surface charge.

When a metal is exposed to an electric field, conduction electrons are displaced within a femtosecond timescale to counteract the presence of the perturbing electric field inside the conductor, thus leaving behind immobile positively charged ions (holes). The spatial extent (i.e. skin depth), ζ, over which this field is screened depends on the frequency of the applied electric field. For a DC electric field, due to the abrupt surface discontinuity of the normal component of the displacement field and the high density of conduction electrons, an infinitesimal conductivity change occurs within the first few atomic layers. As such, the phenomenon could only be observed at a high field *E* = 10^6^–10^8^ V/cm, and for a metal thickness of *d* ≤ Λ, where Λ is the electron’s mean free path. At these extreme conditions, ballistic electron transport and scattering from *d*-level states dominate the electron transport^6^. Furthermore, size effects, ascribed to grain boundary scattering, produce noticeable deviations from the conduction bulk behavior^7^,^8^,^9^. However, for time-varying electric fields, the scenario is less restrictive. Here, the field screening length scale, ζ(ω) (i.e. skin depth), ranges from 10–100 nm. As charges accumulate within ζ(ω), the induced surface charge density, *ρ*_*s*_, modifies the electronic density of states. Accordingly, the non-equilibrium surface charge accumulation maps itself as a local change in the metal’s surface conductivity, σ_s_(ω).

Owing to the large density of background electrons, the high magnitude of the electric field required, chemical bonding, surface potential presence, field-assisted electron grain boundary hopping, and the distortion of the metallic band structure due to charge transfer between the metal film and the supporting substrate, it remains a challenge to isolate the electric field-induced conduction from other dominate contributing backgrounds. Nonetheless, these can be overcome by exploiting terahertz (THz) plasmonic field interaction, which has provided a stimulating domain for studying conduction electron interaction in metals such as photonic magnetoresistance^10^, spinplasmonic electron transport^11^, metal-metal contact resistance^12^, and Schottky barrier conduction^13^. THz conductivity modulation has also been observed in optically pumped Au-Si metamaterials^14^.

In this Letter, we report on experimental observation of THz field-induced conductivity modulation in a metallic gold plasmonic medium using phase-controlled THz-pump/THz-probe time-resolved transmission spectroscopy. By virtue of the metal’s high THz frequency permittivity, 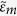, and plasmonic near-field enhancement, ultrafast intensified THz radiation transmission modulation is observed at a low THz field of 12 V/cm. The enhanced THz radiation energy transport is attributed to the presence of induced surface charges and to the large surface-to-volume near-field interaction region provided by the localized THz plasmonic field. Evidence of the coherence of the phenomenon is revealed via in-phase and out-of-phase THz-pump/THz-probe time-resolved spectroscopy. These findings offer a new platform for studying charge dynamics in metallic surfaces, and open the door to technological applications and fundamental studies of new photonic materials based on ultrafast all-THz plasmonic field modulation of metallic structures.

The key aspects in observing such a weak phenomenon are: (*i*) modifying the electronic density of states in ζ by having a high surface-to-volume ratio to affect σ_s_(ω), and (*ii*) ensuring that the applied field is below the breakdown strength or field emission threshold. To satisfy these criteria, a THz plasmonic near-field coupling to a sub-wavelength size metallic surface is employed. By taking advantage of the large ζ ≈ 90 nm (Ref. 15), and field enhancement, an augmented Δσ_s_(ω) signature can be realized.

The interaction of metals with electromagnetic radiation is dictated by the free conduction electrons at the surface of the metal. Consider a single spherical metallic particle of radius *R*  (*R*    l_THz_) placed in free space of permittivity *ε*_0_. Within the framework of the quasi-static approximation, a time-varying THz field, ***E***(***r***,*t*) = Re{***E***_0_(***r***,*ω*)*e*^−*iωt*^} polarizes the particle to induce a time-varying Hertzian dipole moment^16^ (see Supplementary Fig. S1 online):


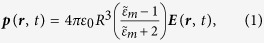


Accordingly, the conduction electrons current density, ***J***(***r***,*t*) = Re{***J***_0_(***r***,*ω*)*e*^−*iωt*^}, in ζ(ω) is governed by,


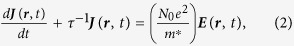


and one customarily writes the current density as: 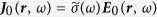.

Where


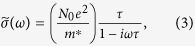


is the complex conductivity, *τ*, *e* and m^*^ are the electron relaxation time, the electron charge and the effective mass, respectively. Within the region ζ(ω) of the polarized metallic particle, the induced current density is dependent on 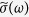 and hence, the radiated *plasmonic* depolarization electric field, ***E***_*pl*_(***r***,*t*), from the oscillating dipole moment, ***p***(***r***,*t*) is given by,


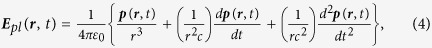


where *c* is the speed of light in vacuum. The terms 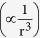, 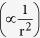, and 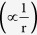 in eq. 4 represent the near, the intermediate, and the far-fields, respectively. Clearly, since 
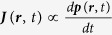
, the radiated far-field THz field strongly depends on 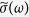.

When the metallic surface is subjected to an additional weaker THz electric field, ***E***^*a*^(***r***,*t*) = Re{***E***^*a*^(***r***,*ω*)*e*^−*iωt*^}, the effective conductivity, 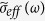, becomes the sum of 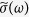 and the additional conductivity due to the induced surface charge density, *ρ*_*s*_, by the perturbing electric field, ***E***^*a*^(***r***,*t*). This effect is further enhanced in an ensemble of sub-λ size metallic particles where the surface electric field enhancement increases from 3× for a single particle to 85× for the ensemble (see Supplementary Fig. S1 online). This enhancement is due to spoof plasmons in micro- and nano-cavities that exist between particles.

## Results & Discussion

To measure the change in conductivity due to induced charges, a sample consisting of three layers of randomly distributed and closely-packed 99.99% pure Au microparticles having a mean diameter of ~100 μm is placed on top of a thin THz-transparent polystyrene substrate. The choice of such plasmonic configuration combined with THz probe radiation has been shown to be sensitive to small changes in conductivity^10^,^11^,^12^,^13^. The THz-pump:THz-probe (THZ-PP) experimental setup used is described elsewhere^17^. Briefly, it consists of two independent photoconductive THz radiation emitters that are synchronized to be either in- or out- of phase. The Au sample is placed at the 0.8 mm focal spot of the THz-PP setup and is excited by a THz pump pulse with an electric field (***E***(*t*)) magnitude of 12 V/cm (see Supplementary Material online for electric field strength calculation) and pulse duration of 1 ps. To ensure no coupling between ***E***(*t*) and the probe electric field pulse, (***E***^*a*^(*t*)), throughout the experiment, the ratio 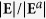 is set at 10:1. The THz field-induced charge effect on particle plasmon formation is recorded as a function of the THz pump-probe pulses relative delay time, *∆t*, by lock-in detection of the time-dependent THz transmission of the radiated THz plasmonic field, 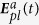, resulting from the coupling of the probe ***E***^*a*^(*t*) pulse to the ensemble. The transport of THz electromagnetic energy through this ensemble of particles is governed by nearest-neighbour plasmonic field coupling and has been studied extensively through THz radiation transmission in a variety of different media^10^,^11^,^12^,^13^,^15^,^16^,^17^,^18^,^19^,^20^,^21^,^22^,^23^. It should be noted that the THz pump and THz probe pulses are modulated at different frequencies, allowing for lock-in detection of only the radiated THz 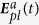 probe pulse electric field. Such a versatile THz pump-THz probe experimental setup has been extensively characterized in reference 17, where the detected THz probe pulse electric field can be selectively isolated and measured with a signal to noise ratio of 10,000:1. Since the degree of THz 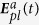 probe pulse electric field signal acquired at the modulation frequency of the THz ***E***^*a*^(*t*) probe pulse is directly proportional to 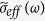 of the Au sample, any change in transmission due to THz pump pulse ***E***(*t*) can only be attributed to the induced *ρ*_*s*_ by the THz pump pulse electric field.

**Figure 1:**
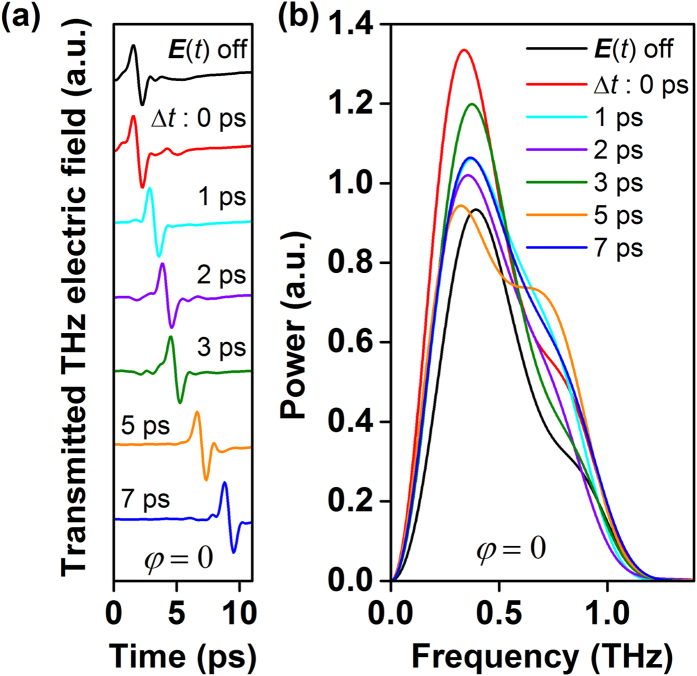
(a) In-phase (*φ* = 0) time-domain reradiated THz probe electric field signals transmitted through the plasmonic sample at various delay times (∆t). (**b**) The corresponding spectral power plots at various delay times (∆t).

Figure 1(a) depicts the time-domain electric field of the THz probe pulse transmitted through the Au particles at various time delays (*∆t* = 0, 1, 2, 3, 5 and 7 ps) along with the case, at ∆t = 0, when there is no THz ***E***(*t*) pump pulse.

Here, the probe electric field, ***E***^*a*^(*t*) is set to be in-phase (*φ* = 0) with ***E***(*t*). Interestingly, examining the transmitted THz 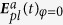 waveforms, for all delays, reveals that their amplitudes are higher than when the THz pump pulse is turned off. The 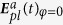 transmission enhancement is better illustrated by comparing the change in the pulse’s spectral power density for each time-resolved signal. As shown in Fig. 1(b), the transmitted THz probe pulse power is lowest when there is no THz pump pulse and the shape of its spectral content is modified in the presence of ***E***(*t*). The transmitted THz probe pulse power is the highest at *∆t* = 0, when the THz probe pulse interacts with the sample at the same time as the THz pump pulse. The spectral reshaping is an indication that the presence of charges induced by the THz pump pulse and their time dynamics act to distort the phase of the probe THz pulse. Minor spectral reshaping will also be present based on the size and structure of the Au particles themselves.

**Figure 2:**
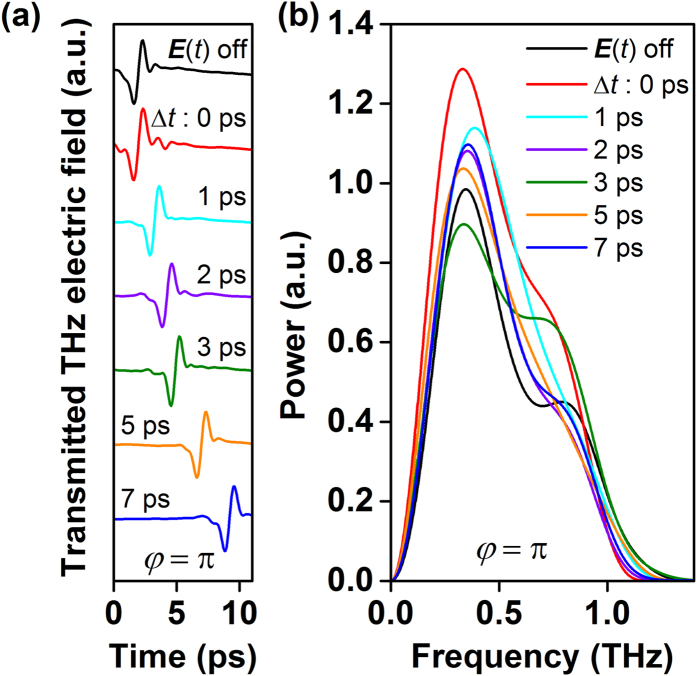
(a) Out-of-phase (*φ* = π) time-domain reradiated THz probe electric field signals transmitted through the plasmonic sample at various delay times (∆t). (**b**) The corresponding spectral power plots at various delay times (∆t).

While changing the relative *∆t* between the pump and probe THz pulses and detecting 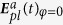 effectively samples the surface charge effects as a function of time, such time-sampling process does not permit probing 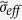 at various phases of the time-domain THz ***E***(*t*) field cycles since the two THz fields have the same duration and the electrons are induced almost instantaneously within a few femtoseconds by the THz ***E***(*t*) field. Nonetheless, the phase-dependency of the observed phenomenon can be distinguished at *∆t* = 0 where ***E***^*a*^(*t*) traverses and probes, in a travelling wave fashion, the sample at the same instant and locations where electrons are induced by the THz ***E***(*t*) field. To ascertain this premise, the ***E***^*a*^(*t*) probe pulse electric field polarity is reversed such that it is out-of phase (*φ* = π) with respect to ***E***(*t*) pump pulse^17^. As shown in Fig. 2(a,b), similar to the *φ* = 0 situation, the 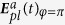 waveform amplitudes and spectral powers are higher in the presence of ***E***(*t*) pump pulse; though, the degree of enhancement is less than that for ***E***^*a*^(*t*)_*φ*=0_. This suggests that even though, in both situations, the same amount *ρ*_*s*_ is induced by ***E***(*t*) (amounting to the same 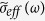), the coupling of ***E***^*a*^(*t*) to the ensemble and the formation of the particle plasmon currents is sensitive to the phase dynamics of the induced *ρ*_*s*._

To estimate the change in the conductivity, we invoke the quasi-static approximation for a polarized metallic sphere. At the surface boundary between metal and free space (*r* = *R*), *ρ*_*s*_ can be evaluated at any point on the surface by the discontinuity of the normal components of the electric flux densities to obtain:


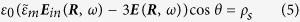


where ***E***_*in*_(***R***,*ω*) is the THz electric field inside the metal surface and *θ* is the angle between the fields and normal unit vector. We calculate the relative magnitude ***E***_*in*_(***R***,*ω*)/***E***(***R***,*ω*) to be ~0.95 within 5 nm from the surface^15^. For ***E***(***R***,*ω*) = 12 V/cm, with a plasmonic field enhancement of ≈85 at *R* (see Supplementary Fig. S1 online), and 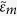 ≈−1.12 × 10^5^ + *i* 7.22 × 10^5^ (Ref. 24), we estimate *ρ*_*s*_ = 1 × 10^−5^ C/cm^2^. Notably, this value is comparable to Stadler’s estimation of 4.8 × 10^−5^ C/cm^2^ (Ref. 5). At this THz electric field magnitude, we estimate our Δσ_s_(ω) to be 1%.

**Figure 3:**
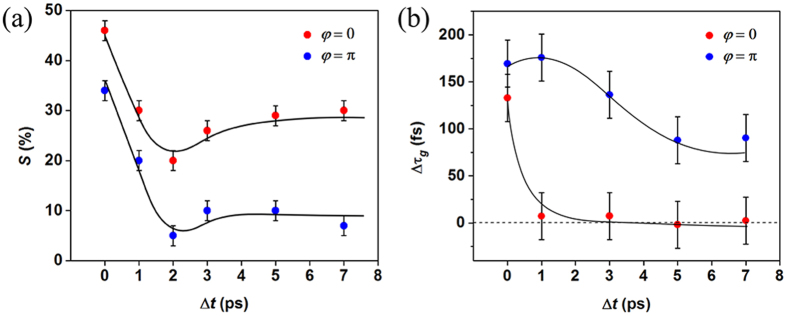
(a) Normalized integrated spectral power as a function of various delay times (∆t) for in-phase and out-of-phase THz probe electric field pulse. (**b**) Frequency-average group delay difference as a function of various delay times (∆t) for in-phase and out-of-phase THz probe electric field pulse. Note that the black lines are inserted to guide the eye.

To further quantify the THz field induced effect on the THz probe pulse, Fig. 3(a) depicts the percent change of the integrated spectral power at each *∆t*, 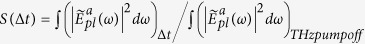, normalized to the integrated spectral power in the absence of the THz pump pulse. Remarkably, for *φ* = 0, at *∆t* = 0, *S* is measured to be 46% higher than when the THz ***E***(*t*) pump pulse is absent. The transmission enhancement is reduced at *∆t* = 2 ps to a minimum of 20% and increases again, for *∆t* ≥ 3 ps, to reach a constant steady state value of 27%. While *S* at *φ* = 0 and *φ* = π exhibits a similar trend for all *∆t*, there is a notable offset of ~15% between the two curves. For *φ* = π, at ∆t = 0, *S* is greatly reduced to 34%, where at *∆t* = 2 ps, it exhibits a minimum of 5% and for *∆t* ≥ 3 ps, a steady state value of 10% is reached.

In the absence of the ***E***(*t*) pump pulse, the free surface electrons within the ζ(ω) layer carry the plasmonic conduction currents from the probe ***E***^*a*^(*t*) pulse. Here, 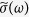 is constant and is determined by the background electron density *N*_0_ in the metal; making the near-field coupling efficiency between ***E***^*a*^(*t*) and the radiated THz plasmonic field, 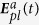, conductivity-dependent. However, in the presence of the pump ***E***(*t*) electric field, additional electrons are drawn into the ζ(ω) region at the surface to screen ***E***(*t*). Within a few femtoseconds, the electrons distribute themselves over the metallic surface in accordance with the local ***E***(*t*) magnitude. This distribution is spatially nonuniform, being higher at the gaps between adjacent particles and at localized spots at surface crevices.

When the probe ***E***^*a*^(*t*)_*φ*=0_ field is in-phase with the ***E***(*t*) field, its induced electrons are displaced also in-phase with the electrons induced and driven by the pump ***E***(*t*) electric field. Collectively, the presence of additional coherently-driven electrons in the ζ(ω) layer makes a notable increase to 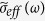 (or accordingly the surface plasmon currents) and thus the radiated THz 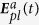 field. In particular, at a delay of *∆t* = 0 ps, where the two THz fields are phase-synchronized and propagate in a travelling wave manner, the instantaneous-modulation of the local 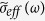 is *sampled* locally by the ***E***^*a*^(*t*)_*φ*=0_ probe field pulse as enhanced surface conductance. This is evidenced as the maximum contribution to the 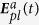 field occurs at this time where the induced background conductivity is coherently oscillating with the probing ***E***^*a*^(*t*)_*φ*=0_. However, when ***E***^*a*^(*t*)_*φ*=*π*_ is out of-phase with ***E***(*t*), whilst the same number of electrons are induced by both THz fields as in the *φ* = 0 THz pump-probe arrangement, the induced electrons due to ***E***^*a*^(*t*)_*φ*=*π*_ are displaced out of-phase with those induced by the ***E***(*t*) field. Effectively, the local conductivity in ζ(ω) is still enhanced by the additional electrons; however, since these are oscillating out of-phase, they introduce more damping on the oscillating plasmonic currents. As such the radiated 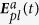 far-field is expected to be lower than the *φ* = 0 case.

Through this picture of the physical processes involved, the transmission enhancement of the probe pulse with the presence of the THz pump pulse can be described for all delays. At 0 < *∆t* < 1.7 ps and for both cases when *φ* = 0 and *φ* = *π*, the ***E***^*a*^(*t*) probes 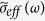 at various phases. This is evident by the observed similarity between *S*(Δ*t*)_*φ*=0_ and *S*(Δ*t*)_*φ*=π_. For *φ* = 0 and at *∆t* = 0.7 ps, the probe ***E***^*a*^(*t*)_*φ*=0_ positive field half cycle overlaps with the negative field half cycle of the pump ***E***(*t*) creating a situation equivalent to the *φ* = *π* pump-probe configuration at *∆t* = 0 where the sample interacts with a unipolar field that dampens the oscillating plasmonic currents. As evidenced from Fig. 3(a), *S*(Δ*t* = 0.7*ps*)_*φ*=0_ ≈ *S*(Δ*t* = 0*ps*)_*φ*=π_. Since the propagation speed of the ***E***(*t*) pump electric field pulse inside the sample is ≈0.62c (Refs. 18,19) and the effective sample thickness is ≈300 μm (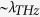), the main ***E***(*t*) pulse traverses the entire sample and exits within ~1.7 ps. Thus, for delay times >1.7 ps, the probe ***E***^*a*^(*t*) pulse sojourns and samples charge distribution due to circulating plasmonic surface currents and reflections from the end face. These plasmonic surface currents persist for ~20 ps, during which the induced charges spread through the entire sample, as such the *S*(Δ*t* > 7*ps*)_*φ*=0,π_ exhibits a steady state value.

Examining the time-domain 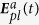 pulse reveals a noticeable pulse reshaping of the electric field signal in the presence of ***E***(*t*). Any change associated with the imaginary part of the impedance of the system is contained in the phase of the transmitted THz pulse. The ascribed relative phase distortion, ΔΩ(*ω*) (relative to ***E***(*t*) being off), represents the time-dependent *ρ*_*s*_ induced dispersion in the plasmonic ensemble. An accurate parameter influenced by ΔΩ(*ω*), is the frequency-average group delay difference, 
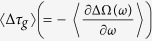
, which quantifies the average retardation time for 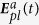 emission from the whole polarized ensemble. The exact shape of the 〈Δ*τ*_*g*_〉 curve depends on the polarity and where the charges are induced in the ensemble. As depicted in Fig. 3(b), for 〈Δ*τ*_*g*_〉 at *φ* = 0, the only significant 〈Δ*τ*_*g*_〉 (=140 fs), occurs at Δ*t* = 0, reaffirming that the induced background *ρ*_*s*_ spatial distribution in the sample readjusts within ~140 fs in response to the 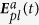 oscillations. At this time delay (i.e. Δ*t* = 0), a maximum THz field transmission is observed. At delays of Δ*t* > 0, 〈Δ*τ*_*g*_〉≈0, indicating that *ρ*_*s*_ in the ensemble relaxes to a steady state which oscillates in unison with ***p***(***r**,t*). However, the situation is strikingly different for *φ* = π. While it takes a slightly longer time (〈Δ*τ*_*g*_〉 = 170 fs) for the induced background *ρ*_*s*_ to redistribute in response to the 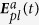 oscillations at Δ = 0 and for Δ*t* > 0, the emission is delayed by several picoseconds. As *ρ*_*s*_ oscillates in opposite phase with respect to 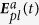, and at Δ*t* > 0, the THz probe field experiences regions where the conductivity varies both in time and space along the path traversed. Each conductivity region can be oscillating in-phase or out of phase with the probe field. As such, the redistribution of *ρ*_*s*_ can only reach steady state when the entire ensemble oscillates collectively in unison where all of the ***p***(***r***,*t*) are coherently-coupled. However, the time to reach this state corresponds to the sojourn time of 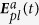 which is several picoseconds. This leads to a lower emission efficiency, as observed in *S*(Δ*t*)_*φ*=*π*_, in Fig. 3(a).

## Conclusion

Now that a new electric field-dependent plasmon transport phenomenon has been revealed, a novel avenue has been opened in terahertz plasmonics. It is envisioned that with the ability to utilize an ultrafast electric field to modulate metal’s surface conductivity and manipulate near-field mediated light transport promises a unique degree of freedom in the development of light-based information devices.

## Methods

### THz pump-probe time-domain spectroscopy system

A THz pump-THz probe time-domain spectroscopy system was employed to measure the transmission through the ensemble of Au particles. It consists of two independently addressable photoconductive (PC) THz emitters. The PC THz emitters consist of 2 mm long, 10 μm wide Cr/Au (20 nm/100 nm) coplanar transmission lines with a 70 μm gap fabricated on a 500 μm thick GaAs substrate. Each THz emitter is independently biased with a 20 Vpp square wave at frequency *f*_*1*_ (pump) and *f*_*2*_ (probe). The THz emitters are excited by 10 fs, λ = 800 nm laser pulses from a Ti:Sapphire laser oscillator at a repetition rate of 80 MHz. The THz time domain signal is acquired via electro-optic sampling in a 500 μm thick <111> ZnSe electro-optic crystal by collecting the THz radiation and co-linearly focusing it with an optical probe pulse (λ = 800 nm) on the ZnSe crystal. A balanced photodetection setup consisting of a quarter-wave plate, Wollaston prism and a balanced photodetector (New Focus Nirvana Detector Model 2007) extracts the THz induced polarization modulation of the optical probe pulse, and lock-in detection is carried out at the bias voltage modulation frequency of the THz probe pulse (*f*_*2*_) with a lock-in amplifier (Stanford Research Systems SR830). This system has been extensively characterized to verify that there are no non-linear effects or harmonics that would result in incorrect measurements^17^. Operating the system at the worst case scenario of *f*_*1*_ = 2×*f*_*2*_, this setup is able to achieve a signal to noise ratio of greater than 10^4^:1. Further details of the experimental setup can be found in Ref. 17.

## Additional Information

**How to cite this article**: Elezzabi, A. Y. *et al*. Terahertz Plasmonic Field-Induced Conductivity Modulation in Gold. *Sci. Rep*. **5**, 10812; doi: 10.1038/srep10812 (2015).

## Supplementary Material

Supplementary Information
