# Supplementary material for: Terahertz Plasmonic Field-Induced Conductivity Modulation in Gold
Source: Sci Rep. 2015 Jun 10;5:10812. doi: 10.1038/srep10812 (PMC4462029; doi:10.1038/srep10812)
Supplement: Supplementary Information [file srep10812-s1.pdf]

Supplemental Material:
Terahertz Plasmonic Field-Induced Conductivity Modulation in Gold

A. Y. Elezzabi, P. Maraghechi and S.R. Greig*
Ultrafast Optics and Nano-Photonics Laboratory
Department of Electrical and Computer Engineering
University of Alberta
Edmonton, AB
T6G 2V4 Canada

*Correspondence to sgreig@ualberta.ca

Calculation of local electric field enhancement

The THz plasmon current dynamics, localized plasmonic field strength, and the radiated depolarization THz plasmonic field (i.e. $\boldsymbol{E}_{pl}^{a}(\boldsymbol{r},t)$ due to $\boldsymbol{E}^{a}(\boldsymbol{r},t)$ coupling), are strongly influenced by collective surface conductivity, the interparticle separation, s, and the THz plasmon field evanescent decay distance, ℓ. For $s/\ell > 1$, the interparticle interaction is governed by far-field dipolar or multipolar absorption and reradiation, whereas, for closely packed metallic particles $s/\ell << 1$, high evanescent fields result in a strong near-field coupling [1]. It is evident that any changes in $\tilde{\sigma}$ due to electric-field induced charges will map itself on the radiated THz electric field.

Using finite difference time domain calculations, we show that in an ensemble of sub-λ size metallic particles the surface electric field enhancement increases from 3× for a single particle to 85× for the ensemble, as shown in Fig. S1. Thus, the incident 15V/cm THz electric field is enhanced to 1.275 kV/cm within the 10nm gap between the particles.

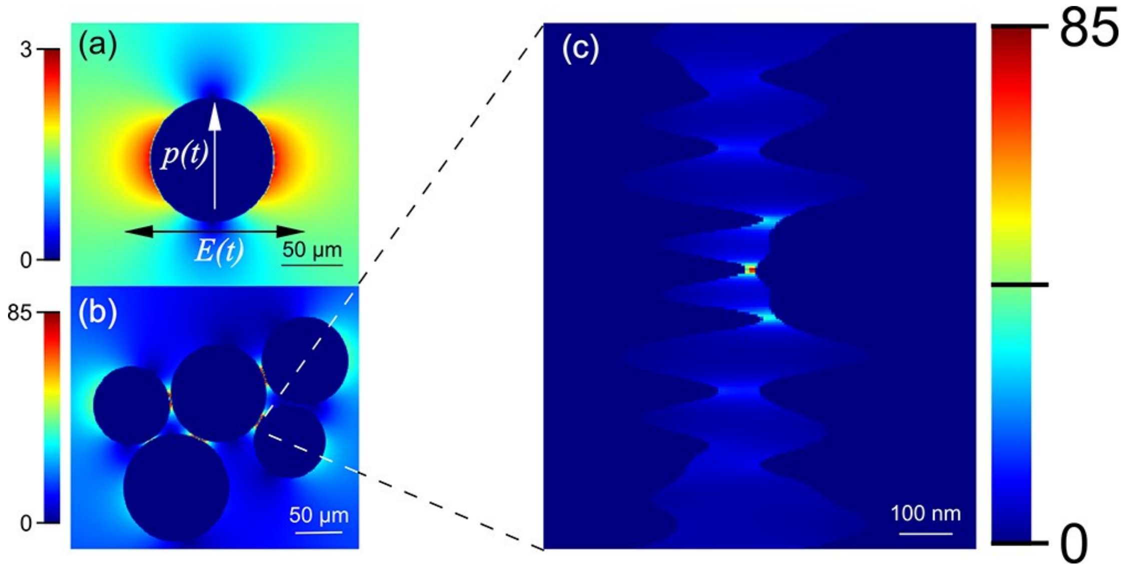

FIG. S1. (a) Surface electric field enhancement for a single particle excited with $\boldsymbol{E}(\boldsymbol{t})$. The two-headed arrow represents the polarization direction of the incident THz electric field and $\boldsymbol{p}(\boldsymbol{t})$ represents the induced Hertzian dipole moment. (b) Surface electric field enhancement for an ensemble of particles showing inter-particle THz field enhancement of 85 times. (c) Enlarged image showing the electric field enhancement between two particles.

Estimation of the THz pump electric field strength

The THz pump pulse used in these experiments is generated from a 70 μm gap photoconductive stripline antenna patterned on a LT-GaAs substrate biased with 20V (~2.86 kV/cm) and illuminated with a 60 mW, 10 fs, λ = 800 nm optical pulse

The magnitude of the THz pump electric field can be estimated from the differential power of the optical probe beam at the input to the balanced photodetector, which can be obtained from the

time domain signal detected by the lock-in amplifier. Knowing this, the THz pump electric field can be estimated via the following equation:

$$E_{THz} = 2\frac{\Delta P}{P_{probe}}\sqrt{\frac{3}{8}}\frac{c}{2\pi\nu n^3 r_{41} L}$$

Where:

ΔP = differential power at the photodetector (54 nW)

P_{probe} = power of the optical probe pulse (0.5 mW)

c = speed of light in vacuum

ν = frequency of optical probe pulse (375 THz)

n = refractive index of ZnSe electro-optic crystal (2.5295)
r_{41} = electro-optic coefficient of ZnSe (2 pm/V)

L = thickness of ZnSe crystal (500 μm)

With these parameters, and this equation, the THz pump electric field is estimated to be 10 V/cm.

Additionally, the THz electric field strength can also be estimated from the conversion efficiency of our photoconductive antenna. Here, the THz electric field strength at the focus is given by:

$$E_{THz} = \sqrt{\frac{2\eta P_{optical}}{f_{rep}\tau_{THz}\varepsilon_0 c\pi x_f y_f}}$$

Where:

η = optical to THz conversion efficiency (5×10^{-6})

$P_{optical}$ = average power of the optical pump pulse (60 mW)

f_{rep} = repetition rate of the laser (80 MHz)

τ_{THz} = pulse length of the THz pump pulse (1 ps)

ε_0 = permittivity of free space

c = speed of light in vacuum

x_f = full-width-at-half-maximum of the THz focal region in the x direction (510 μm)

y_f = full-width-at-half-maximum of the THz focal region in the y direction (740 μm)

To estimate η, we compare our geometry to that of a similar geometry with a known conversion efficiency [2]. The geometry employed in [2] is that of an 80 μm gap stripline antenna patterned on LT-GaAs and biased with 100 V, leading to a bias electric field strength of 12.5 kV/cm. A THz pulse of 0.09 μW is produced from this geometry when illuminated by a 15 mW optical pulse, signifying a conversion efficiency of 6×10^{-6}. As the THz power scales quadratically with both bias electric field strength and optical pump power [2], our bias electric field strength is 4.37× lower than [2], and our optical pump power is 4× higher than [2], we estimate our conversion efficiency to be 5×10^{-6}. Utilizing this conversion efficiency as well as the other parameters for our setup [3], the estimated THz electric field strength at the focus is found to be 15 V/cm.

Based on these two estimations (upper and lower limits), we arrive at a THz pump electric field strength of 12 V/cm.

[1] K. J. Chau, G. D. Dice, and A. Y. Elezzabi, Phys. Rev. Lett., **94**, 173904 (2005).
[2] M. Tani, S. Matsuura, K. Sakai, and S-I Nakashima, Appl. Optics, **36**, 7853 (1997).
[3] A. Y. Elezzabi, and P. Maraghechi, *Rev. Sci. Instrum.* **83,** 053107, (2012).
